# Microtubules, Myelin Sheaths, and Altered Behavior

**DOI:** 10.1523/ENEURO.0520-20.2020

**Published:** 2021-01-27

**Authors:** Alexia Crockett

**Affiliations:** Department of Biological Sciences, University of South Carolina, Columbia, SC 29208

Mice are skttish creatures that innately hide from perceived threats by staying out of open areas, freezing to avoid predators, or hiding in dark spaces when an adverse stimulus is sensed. However, after inducing a global TPPP (tubulin polymerization promoting protein) gene knock-out (KO), which results in reduced myelin length and thickness (Fu et al., 2019), Nguyen et al. (2020) found that these natural behaviors become less pronounced (Fig. 1). They also report that fear conditioning is less effective in the *Tppp* KO mice, indicating that their ability to learn to fear a specific event is impaired. Fear impacts many brain circuits and is critical for survival, but the contribution of non-neuronal cells in fear circuits is not well understood. This work identifies a protein that influences both innate and learned fear via its role in microtubule nucleation in oligodendrocytes.

**Figure 1.**
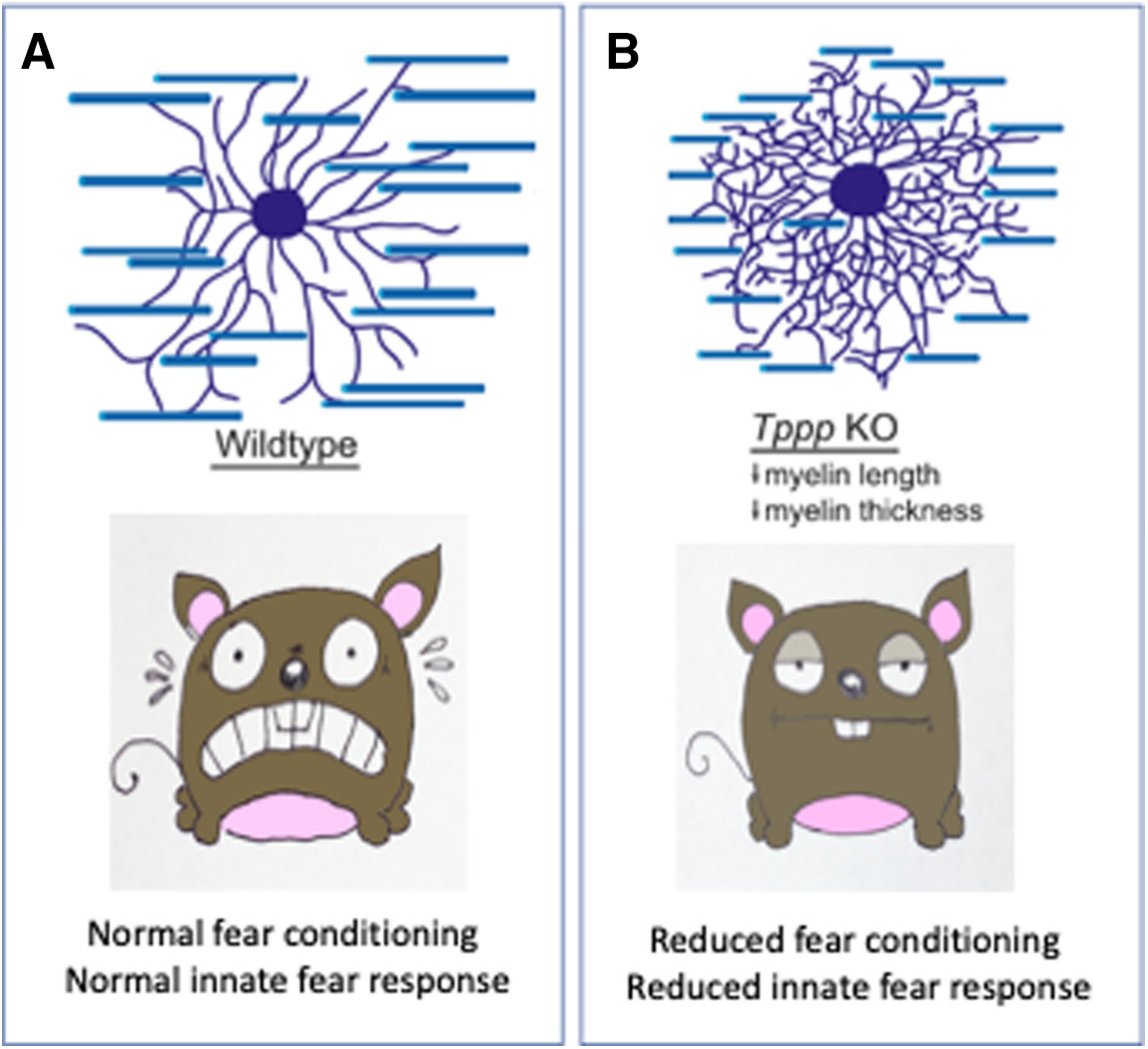
***A***) Normal branching and myelin sheath length in oligodendrocytes in WT mice is coupled with normal fear conditioning and innate fear responses. ***B***) *Tppp* KO results in highly branched oligodendrocytes with shorter, thinner myelin sheaths. These mice have dramatically reduced fear conditioning and innate fear responses.

In the cell body of most cells, microtubules are nucleated from perinuclear centrosomes. These microtubule organizing centers (MTOCs) contain centrioles and a pericentriolar matrix containing g-tubulin ring complexes (γTURCs). γTURCs are the starting point for the initiation and subsequent polymerization of α/βtubulin dimers into cytoplasmic microtubules. Golgi outposts are a different type of MTOC that nucleate microtubules outside of the cell body in several cell types (Valenzuela et al., 2020). TPPP is enriched at Golgi outposts and serves as a marker for these structures (Fu et al., 2019). TPPP is highly expressed in oligodendrocytes, but the expression is extremely low in other brain cells (Zhang et al., 2014) or non-neural tissues (Schaum et al., 2018). The behavioral impacts of a global *Tppp* KO are likely related to TPPP’s involvement in myelin structure (Fu et al., 2019). Myelin is an axonal insulator produced by oligodendrocytes that enables saltatory conduction of action potentials. Communication between brain regions often depends on white matter tracts containing axons and the surrounding myelin. Defects in myelin can cause time delays and disrupt the structural connectome. In oligodendrocytes, microtubules can be classified as radial, which are near cell bodies and extend toward the axon, or lamellar, which initiate farther away from the cell bodies and spiral around the myelin sheath. These radial microtubules contribute to myelin elongation. Oligodendrocytes in which TPPP was depleted had shorter lamellar microtubules resulting in short, thin myelin sheaths (Fu et al., 2019). Cultured *Tppp* KO oligodendrocytes were found to have more proximal branches, mixed microtubule polarity, and higher accumulation of myelin basic protein mRNA. Myelin was also strikingly reduced in many brain areas. These findings point to an essential role for lamellar microtubules in myelination. Myelin changes were observed at postnatal day 14, suggesting altered developmental myelination (Fu et al., 2019). However, myelin maintenance could also be disrupted by TPPP depletion.

Impaired white matter integrity and reduced expression of myelin-related genes has been observed in patients with schizophrenia, a neurodevelopmental disorder characterized by hallucinations, social deficits, and delusions (Takahashi et al., 2011). In mice, excess myelination results in increased fear-conditioned learning, whereas reduced myelination results in inhibition of fear-conditioned learning. Several genes have been linked to these observations, including MYRF (myelin regulatory factor), Cdk5 (cyclin-dependent kinase 5), and ERK1/2 (extracellular signal-regulated kinase); (for specific references, see Nguyen et al., 2020). Transcription factors and kinases can have broad impacts that are not necessarily related to myelin structure. The finding that depletion of TPPP impacts fear-based behaviors is intriguing, because it more directly links tubulin nucleation and the structure of myelin sheaths to fear behaviors.

Interestingly, *Tppp* KO significantly altered behavior in tests designed to assess learned and innate fear (see below) but did not impact behavior in tests evaluating anxiety. The open-field and light-dark box tests are commonly used to observe anxiety-like behaviors. In the open-field test, subjects are placed in a box with intersecting infrared beams along the floor to monitor the overall distance traveled, speed, twirling/spinning, and rearing activities. Any of these behaviors in excess, especially in the box’s central region, indicate anxious behavior. For the light-dark box assay, subjects are placed in a box with separate light and dark regions connected by a small opening. More time spent in the dark region indicates increased anxiety. *Tppp* KO mice displayed normal activity in both assays, demonstrating that hypomyelination in these mice did not alter anxiety compared with control animals.

The most interesting findings in this study came from measuring fear conditioning, spatial memory, and looming fear responses (Fig. 1). During fear conditioning testing, mice were subjected to an auditory cue followed by a foot shock four times. On day 1, both wild-type (WT) and *Tppp* KO mice displayed similar amounts of freezing, whereas on day 2, when context recall was observed, *Tppp* KOs spent half as much time freezing as WTs, demonstrating defective fear conditioning. Decreased freezing responses were also displayed by *Tppp* KOs when introduced to the auditory cue in a new environment with a new olfactory cue on day 3, pointing to defective cue-based recall. To test innate fear, mice were exposed to a looming stimulus, an expanding black disk on a white background, 15 expansions over 24 s, projected overhead on an LCD monitor. The looming shadow caused WT mice to freeze or hide in response. *Tppp* KOs showed significantly decreased hiding, pointing to a deficit in the innate fear response. Because *Tppp* KO animals showed increased ambulation, rearing, and head tilts, the authors reasoned that the looming shadow was being perceived but was not eliciting fear. In support of this, a contralateral pupillary light reflex test showed appropriate dilation, indicating no severe visual impairments. Thus, depletion of TPPP and subsequent hypomyelination leads to problems with both memory-dependent and innate fear responses.

These findings contribute to our understanding of how improper myelination can impact behavior and raise some interesting questions. Hypomyelination was observed in the hippocampus and cortex of *Tppp* KO mice (Fu et al., 2019), but brain circuits associated with fear responses (e.g., the amygdalofugal pathway) were not included in that study. These new findings will undoubtedly drive research in this direction. It will also be interesting to determine whether brain regions that contain characteristically longer myelin sheaths are more severely impacted than other areas. Finally, different mouse models that display myelin defects have widely variable behavioral phenotypes. This could be because of regional differences in protein function or the importance of myelin plasticity. The latter is an emerging principle of oligodendrocyte biology. Linking microtubule dynamics in oligodendrocytes to behavioral deficits opens up new research avenues into the etiology of human disorders like schizophrenia.

## Synthesis

Reviewing Editor: Christophe Bernard, INSERM & Institut de Neurosciences des Systèmes
